# Using Computer Models to Predict Prevention Policy Outcomes

**Published:** 1996

**Authors:** Harold D. Holder

**Affiliations:** Harold D. Holder, Ph.D., is director of and senior scientist at the Prevention Research Center, Berkeley, California

**Keywords:** computer technology, scientific model, prevention program, prevention strategy, program evaluation, arrest, drinking and driving, statistical estimation, risk assessment, traffic accident, public policy on AOD

## Abstract

Computer simulation, or modeling, can illuminate the potential costs and effects of policy alternatives. The SimCom (Simulated Community) model has been under development for more than a decade and has been increasingly successful in simulating the effects of alcohol prevention policy. A recent application of SimCom to a northern California community’s prevention efforts projected the results of an intervention designed to change the perceived risk of arrest for driving under the influence. SimCom simulated the effect of this intervention on the rate of alcohol-involved injury crashes and was able to generate crash estimates for 1993 to 1995 that later closely matched actual data for the same period. Statistical analyses of the northern California (i.e., experimental) site compared with data from a matched comparison site showed significantly fewer crashes in the experimental community. Although the complexity of computer models may present many more data collection, communication, and technical challenges than traditional policy research, with further refinement, computer simulations are likely to become vital components of prevention efforts to reduce alcohol-related problems.

Public policy by nature is prospective (i.e., it anticipates future events); research, however, is primarily retrospective (i.e., it analyzes past events). Public policy to reduce alcohol-involved problems is best formulated when decisionmakers are aware of the potential cost and future effects of each prevention strategy alternative. Projecting policy outcomes is a difficult task, one that must be informed by the best research. The challenge of developing scientifically based prevention policy is particularly difficult at the local level, because most alcohol policy research has been conducted at the national or State level. As a result, local decisionmakers frequently lack community-based scientific evidence on which to build alcohol policy (see Edwards et al. 1994 and Holder and Edwards 1995).

Statistical techniques using retrospective data frequently are used in alcohol research; these methods determine relationships between specific variables (e.g., the connection between the quantity of alcohol consumed and the likelihood of involvement in a traffic crash). However, traditional statistical methods are limited in their ability to inform long-range planning. For example, cross-sectional data analysis techniques, such as those examining the data obtained from a school or community survey, provide only a “snapshot” of a situation. Time-series and longitudinal statistical techniques, which have been used successfully in alcohol policy analyses, provide information about changes over time in specific variables or subjects but nevertheless can only describe past trends (Blose and Holder 1987; Wagenaar and Holder 1991, 1995; Wagenaar 1986). Statistical analyses also do not identify underlying causal relationships or create an understanding of systems producing changes in variables.

A tool is being developed to help build this understanding and allow policymakers to forecast the outcomes of proposed prevention programs: computer simulation, or modeling. This article describes a computer model of the effects of different alcohol-related policies and an application of the model to an actual community prevention program.^1^

## Computer Models

Computer modeling is a unique tool used to express the causal relationships between variables in a complex system. A computer model can examine history (as a means to explain the past). It also can project the effects of changes in particular phenomena, thereby providing answers to the question, “If I implement alcohol prevention strategy X, what is most likely to happen to alcohol consumption?” Decisionmakers in the military, health care management, and industrial sectors have recognized the benefit of computer modeling in making decisions that may affect established conditions or patterns of behavior. Computer modeling as a research and policy evaluation technique has been used for at least three decades to investigate systemwide problems and changes in problem indicators. Likewise, computer models can help develop an understanding of community-based factors underlying alcohol use and abuse (see Levin et al. 1975; Holder and Blose 1983, 1987, 1988; and Holder (in press *a*,*b*).

A computer model consists of a series of mathematical statements, or algorithms, for estimating the probability of a given outcome under specified conditions. The mathematical formula is derived from variables and data based on the best and most current research available. Although conventional multivariate statistical techniques^2^ might be part of the underlying mathematical structure, the results of computer modeling are much more robust than the results obtained with ordinary statistics. Moreover, results from computer modeling can be used to investigate possible alternative futures.

## Intermediate Variables

Alcohol policies can be defined as purposeful environmental actions, activities, efforts, or structural changes intended to reduce the future occurrence of alcohol problems. These efforts, however, rarely cause a change in an alcohol-involved problem directly. Instead, interventions most often affect related phenomena (i.e., intermediate variables), which in turn directly change a problem.

**Figure 1:**
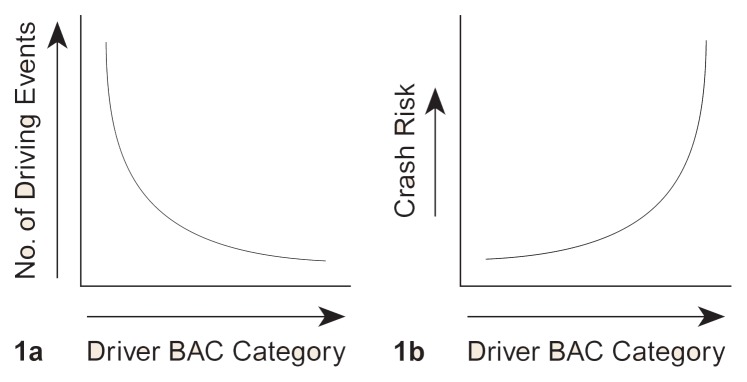
Intermediate variables for alcohol-involved traffic crashes. By combining the distribution (i.e., pattern) of data shown in 1a and 1b, researchers can estimate the number of traffic crashes by driver blood alcohol concentration (BAC) category. Using these and other data, a computer model can predict the number of alcohol-involved traffic crashes over a given period.

For example, an important intermediate variable for alcohol-involved traffic crashes is the total number of driving events over a specific period (e.g., 1 month or 1 year) in which drivers have blood alcohol concentrations (BAC’s) within a designated range. This variable can be thought of as a data pattern (i.e., distribution) consisting of the number of one-way driving trips (i.e., driving events) categorized by driver BAC (figure 1a). On average, driving events involving higher driver BAC’s have a greater crash risk than driving events involving drivers with lower BAC’s, leading to another intermediate variable, crash risk categorized by driver BAC (figure 1b). Combining the actual number of driving events having drivers in particular BAC categories with the crash risk by BAC category generates an estimate for the number of traffic crashes by driver BAC. Using this and other data (e.g., demographic statistics), a computer model can project the number of alcohol-involved traffic crashes in a community over a specific period.

**Figure 2:**
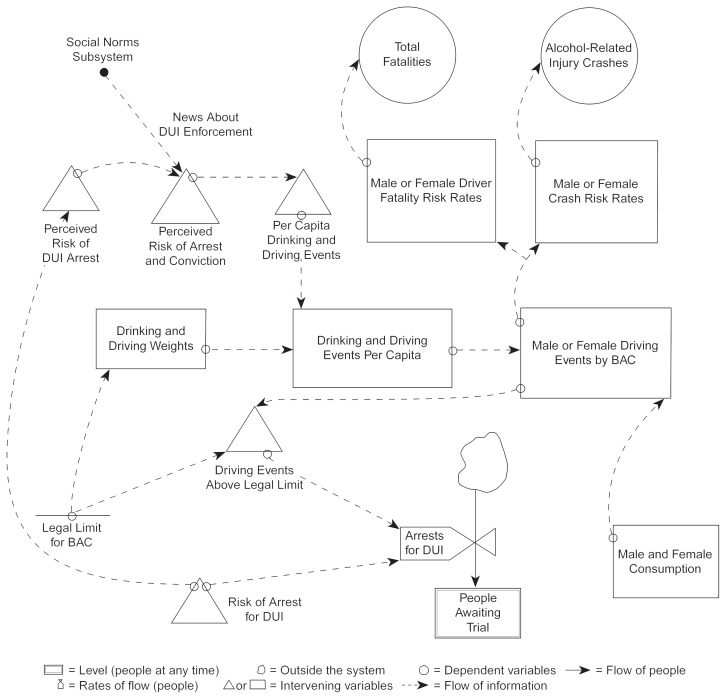
Diagram of selected intermediate variables in the Simulated Community System of Alcohol Use and Abuse (SimCom) computer model. Changes in the perceived risk of arrest for driving under the influence (DUI) affect many other variables.

Intermediate variables both influence and are influenced by other intermediate variables (figure 2); they are interrelated and cannot always be separated into discrete, mutually exclusive categories. As noted above, both the number of driving events by driver BAC and the crash risk by BAC are intermediate causal variables for alcohol-involved traffic crashes. Other examples of intermediate variables that could change the number of alcohol-involved traffic crashes include the following:

- *Public perceptions of the risk of arrest for driving under the influence (DUI)*. This variable, which is related to media attention and actual DUI enforcement, may reduce the number of driving events by drivers with higher BAC’s and thereby lower the number of alcohol-involved crashes.
- *Enforcement of DUI laws*. Increased enforcement activities, such as intensive roadblock checks or the use of special patrols, can deter drinkers who may drive while intoxicated, thereby reducing the number of driving events by drivers with higher BAC’s.
- *The number of driving events*. This variable can be changed, for example, through increases in gasoline prices or reductions in its availability. A gasoline shortage, in turn, could reduce the overall number of driving events and, consequently, the number of driving events in which drivers have been drinking.

When designing prevention and other programs, researchers and prevention specialists look for intermediate variables that can be readily changed to achieve the desired outcome. Programs that change public perceptions of the risk of arrest for DUI are good examples of how intermediate variables become targets of intervention for addressing a problem (in this case, alcohol-involved crashes).

### Perceived Risk of Arrest for DUI as an Intervention Target

Research consistently points to the public’s perceived risk of arrest for DUI as an effective target for interventions to reduce alcohol-involved traffic crashes. For example, Voas and Hause (1987) reported that during a program of special DUI patrols in Stockton, California, the risk of arrest was nearly 10 times greater than average. Alcohol-involved crashes declined after news media drew attention to this increased risk of arrest (i.e., the public’s perception of risk was increased). After the publicity declined, crashes increased, even though the actual risk of arrest remained higher. By the time the 3-year special patrol program was over, the crash rates had returned to their previous levels. Thus, changes in drivers’ perceptions of their risk of arrest altered drinking and driving behavior more than the increase in actual arrest risk did. Likewise, Ross (1982, 1985) has discussed the effectiveness of perceived risk of arrest as a deterrent, noting that the deterrent effect is the result of the certainty of the punishment, not its severity. The threat of immediate mandatory license suspension appears to have a general deterrent effect (Hingson 1993).

## Model Validation

From a generic model of alcohol use and abuse, a unique model for a real community can be developed. Before a model can be used for policy analysis, however, it must be validated. First, the mathematical formulas describing the relationships between the variables are programmed into the computer. Next, the computer is asked to generate data (e.g., the annual number of alcohol-involved crashes) for a specific time period (e.g., 1970–1990) once loaded or initialized, with data for the first year of that time span (i.e., actual data for 1970). The computer’s results are then compared with existing historical data. If the model’s results closely match historical data, the model can be considered a valid representation of the community. If, however, significant differences exist between the model’s results and actual data, the model’s structure and data values are reexamined to locate the source of the problem. Adjustments are made, and the model is tested again. This iterative process helps build understanding of the underlying causal structures. Once a model is validated and the factors (e.g., per capita alcohol consumption) that produce large changes in key system outcomes (e.g., alcohol-problem indicators) are identified, the model can be used to experiment with local prevention policies. Since the model’s results will vary with the level of understanding and the quality of data available, it is possible to determine a level of confidence for the results based on sensitivity analyses. That is, the model is used to test multiple interventions to determine the stability of the estimates. If the results are comparable across interventions where key internal factors are variable, researchers can have more confidence in the model.

**Figure 3:**
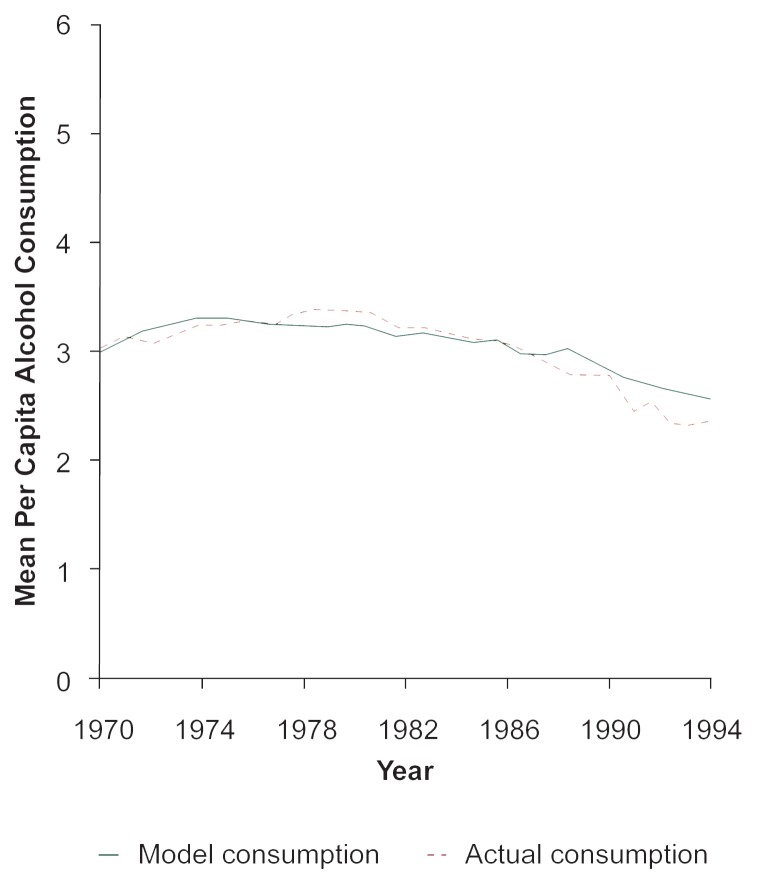
Mean per capita alcohol consumption based on actual data and SimCom projections.

A “good” computer model is one that provides accurate, reliable forecasts as well as an explanation of how factors interact with each other. A “bad” model is one that does not seek to explain or incorporate such interactions, but only statistically projects current or long-term trends. For example, a model predicting alcohol consumption based solely on the national trend between 1970 and 1980 would be incorrect. From 1970 to 1980, the trend was one of increasing consumption; after 1980 consumption levels turned sharply downward and continued to fall for the next 10 years. A good model, when given data for 1970, would be able to generate values for annual alcohol consumption for the period 1970–1990 that matched the actual data over this period, including the upward trend (1970–1980) and the downward trend (1980–1990). (See figure 3.) As with classical experimentation, research using computer simulation involves introducing specific changes (i.e., treatments) into the situation to observe the outcome. Testing alcohol policy with computer simulation thus identifies and alters specific community-based variables to project the outcome of a given prevention policy. Computer models “act like,” or simulate, the actual community to produce information about the future changes likely to be associated with specific alcohol prevention policies.

## SimCom: A Computer Model of Alcohol Use and Abuse

The Prevention Research Center in Berkeley, California, has developed and tested a computer model of the community system of alcohol use and abuse called SimCom (Simulated Community System of Alcohol Use and Abuse). SimCom’s primary uses are as follows: (1) to investigate the complex interrelationships among variables that together explain alcohol use and alcohol-related problems within a specific community and (2) to demonstrate how specific prevention interventions can alter alcohol-use patterns and resulting alcohol-related problems. (For a history of the development of SimCom, see sidebar, p. 259.)

**The History of SimCom**

The first generation of SimCom, a computer model of alcohol use and abuse, was designed and tested between 1980 and 1986 under a National Institute on Alcohol Abuse and Alcoholism (NIAAA) research grant. The first version attempted to recreate changes in U.S. alcohol consumption patterns over the 20-year period from 1960 to 1979. Data used included a breakdown of the U.S. population by age, gender, and alcohol consumption; initial consumption stimulus factors (e.g., alcoholic beverage prices); and changes in the stimulus factors over the period (Holder and Blose 1987). Comparing the model predictions for each year with actual data suggested that only minor differences existed between model results and actual historical data.

The first-generation SimCom then was programmed and given a starting point for calculations (i.e., initialized) using local data from three counties chosen for their variety of social and economic conditions, as well as for their diversity in alcoholic beverage control legislation. Wake County, North Carolina, is a mixed urban-rural county with a population exceeding 300,000 in a State that allows local counties to decide whether alcohol will be sold within the county. Washington County, Vermont, is a small, rural county of 52,000 people in a State that operates a retail alcohol monopoly. Alameda County, California, is a large, urban county with more than one million people in a State that allows sales of alcohol in licensed retail outlets. A unique model of each community was developed using local data; historical data were not loaded into the model but were held aside for benchmark testing.^1^ After the models were successfully benchmarked, researchers then entered data simulating a wide range of prevention interventions (see Holder and Blose 1983, 1987). They used results of the simulations to refine the model.

Further testing and refinement led to a second-generation SimCom model, developed in 1987 for the county of San Diego, California. This version of the model was primarily a policy-testing tool sponsored by the National Highway Traffic Safety Administration. A 17-year period (i.e., 1970 to 1986) was used in benchmark testing the San Diego County model. After the model was successfully tested, leaders of San Diego County identified alcohol-related traffic problems that the community desired to address. A number of prevention alternatives were projected over the period 1987 to 2000: (1) a school-based education program, (2) increased driving-under-the-influence (DUI) enforcement, (3) mandatory license suspension for convicted DUI offenders, (4) mandatory jail for convicted DUI first offenders, (5) lowered legal blood alcohol concentration limits of 0.08 and of 0.05, (6) increased retail price of alcoholic beverages, and (7) increased treatment for multiple-DUI offenders. The changes in alcohol-related traffic injury crashes and alcohol-related driver fatalities were used as annual outcome measures (Holder and Blose 1988). Local decision-makers used the results of these simulations to plan a drinking and driving prevention program. As with the other generations of Sim-Com, researchers refined the model based on differences in actual and projected data.

Researchers continued to hone SimCom, increasingly testing the model in real-world settings rather than in laboratories. The third generation^2^ of the model (1990–1993) was tested by developing comprehensive models for the State of California and two communities, including the County of San Diego. These versions expanded the complexity of the model to add new predictive variables and to allow for more prevention alternatives to be tested. The fourth and fifth generations of SimCom (1993–1995) involved State-level tests of the model in Wisconsin, South Carolina, Hawaii, Kentucky, and Connecticut, as well as in at least one community in each State. Both generations were based on the same general system structure. Each model accurately simulated the real State or community when loaded with specific data from that area.

Each generation of SimCom has produced refinements in the computer model of alcohol use and abuse. With time, researchers hope to produce increasingly accurate versions that can be easily tailored to specific communities.

—Harold D. Holder

ReferencesHolderHDBloseJOPrevention of alcohol-related traffic problems: Computer simulation of alternative strategiesJournal of Safety Research1431151291983HolderHDBloseJOReduction of community alcohol problems: Computer simulation experiments in three countiesJournal of Studies on Alcohol4821241351987356094810.15288/jsa.1987.48.124HolderHDBloseJOCommunity planning and the prevention of alcohol involved traffic problems: An application of computer simulation technologyEvaluation and Program Planning1132672771988

1Benchmark validation compares the actual historical data with model results.2The third and subsequent generations of SimCom were supported by NIAAA national research grants.

## SimCom’s Data Structure

**Figure 4:**
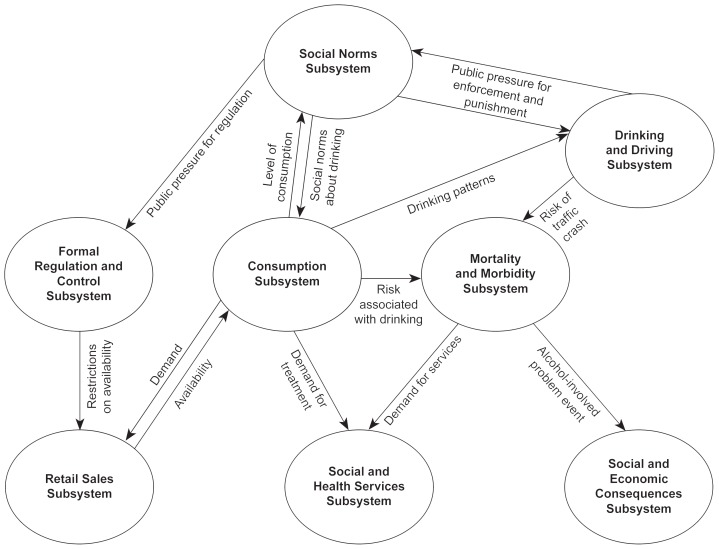
Diagram of the eight interconnected subsystems (i.e., clusters of variables) underlying the SimCom model of community alcohol use and abuse. Arrows indicate the direction of influence of the subsystem (e.g., the consumption subsystem both affects and is affected by the retail sales subsystem).

The variables underlying SimCom are grouped into eight interconnected subsystems (i.e., clusters of related variables) (figure 4). As with all computer models, the subsystems may overlap slightly.

### Consumption Subsystem

The single most critical dynamic in the SimCom model is the shift in patterns of alcohol consumption over time. The model divides the population into four consumption categories according to the average alcohol quantity consumed per day. Each category is separately tracked and modified through the model dynamics. For both genders, 7 age categories are defined, creating a total of 14 categories called age-sex groups (e.g., 18- to 20-year-old males)^3^; people in each age-sex group exhibit similar drinking patterns or are likely to be affected similarly by specific interventions. In the model, changes in drinking behavior are triggered by changes in five variables called stimulation factors: disposable income, alcoholic beverage prices, alcohol availability, social norms, and minimum legal drinking age (MLDA).

### Retail Subsystem

Depending on State alcohol laws, retail establishments may obtain alcohol-sales licenses for on-premises consumption (e.g., bars, pubs, restaurants, or arenas) or for consumption elsewhere (e.g., wine shops, liquor stores, supermarkets, or convenience stores). Sim-Com uses population growth, per capita consumption, and economic indicator data (e.g., average disposable income) to predict the number and types of outlets that are licensed to sell alcoholic beverages.

### Formal Regulation and Control Subsystem

This subsystem simulates the effects of the laws and regulations of State or local regulatory agencies on retail availability of alcohol. Laws and regulations influence alcohol retail sales (e.g., by limiting the number of new liquor licenses) and thereby may affect consumption activity.

### Social Norms Subsystem

In the model, social norms can increase or decrease levels of alcohol consumption, depending on how socially acceptable alcohol use is at a given point in time. Sociocultural determinants of overall community drinking behavior (e.g., the effects of ethnic groups, college students, and military personnel) are also accounted for through this subsystem. A college town in New England, for example, might have higher per capita alcohol consumption than a Southern farming community due to a higher norm or acceptability of drinking. Norms are reflected or indexed in SimCom as a combination of newspaper attention to alcohol problems, actual level of consumption, level of advertising, and any public opinion data. These data are adjusted by racial and ethnic variables in the population.

### Drinking and Driving Subsystem

The number of driving events categorized by driver BAC is determined for the community’s population according to the same age-sex groups described earlier. This pattern is then linked to the number of crashes resulting in driver fatalities and other injuries.

### Mortality and Morbidity Subsystem

This subsystem uses specific risk factors for the different age-sex groups in the community to determine levels of alcohol consumption for each group. Based on these data and other factors, the computer calculates annual numbers or incidences of alcohol-associated deaths, illnesses, and nontraffic injuries.

### Social and Economic Consequences Subsystem

This subsystem models the costs of specific alcohol-related problems, such as alcohol-related fatal crash costs and health care costs.

### Social and Health Services Subsystem

This subsystem reflects the demand for social and health services related to drinking (e.g., the demand for alcohol-related emergency room treatment) and alcoholism treatment. The general approach is to interrelate levels of consumption with risks for alcohol-related problems and the corresponding demand for treatment for each age-sex group. The model permits a community to monitor changes in demand for alcoholism treatment services, general health care services, and social services.

## Evaluating Local Alcohol Policies Through Computer Simulation

### The National Community Trials Project

The Prevention Research Center’s National Community Trials project was established to systematically evaluate a comprehensive, communitywide prevention policy designed to reduce alcohol-involved injuries and death. The project involved six communities: three experimental communities (i.e., those that undertook large-scale preventive interventions) and three matched control communities (i.e., those that conducted no intervention). The experimental communities implemented programs based on five interrelated prevention policy components (see box). Using data gathered from the northern California site (one of the experimental communities), SimCom was used to project the results of the component designed to change one of the intermediate variables described earlier, the perceived risk of arrest for DUI. SimCom simulated the effect that a change in the variable would have on the rate of alcohol-involved injury crashes.^4^

**The National Community Trials Project**

The National Community Trials project, sponsored jointly by the National Institute on Alcohol Abuse and Alcoholism and the Center for Substance Abuse Prevention, involved six communities (three trial [i.e., experimental] communities and three matched control communities). The project’s goal was to reduce alcohol-involved injuries and deaths. Each experimental community was racially and ethnically diverse and had a population of about 100,000; two were in California (one in northern and one in southern California), and one was in South Carolina.

The approach underlying the Community Trials project involved five interactive prevention components:

- The Community Mobilization component focused on developing community organization and support of the project and on increasing public awareness and concern about alcohol-involved trauma.
- The Responsible Beverage Service Practices component included training of servers, owners and managers of bars, clubs, restaurants, and other establishments that serve alcohol in order to reduce intoxicated and underage customers.
- The Reduction of Underage Drinking component included community education on the extent of underage drinking, the training of employees of “off-premises” alcohol retailers (e.g., convenience stores, supermarkets, and package liquor stores), and parental training and mobilization to reduce teenagers’ access to alcohol.
- The Risk of Drinking and Driving component sought to increase efficiency of local enforcement of driving under the influence of alcohol (DUI) and to increase the actual and perceived risk of arrest for DUI.
- The Access to Alcohol component included using local regulatory powers and alcoholic beverage control authority activities to reduce the availability of alcohol (see description of the Community Trials Project in Holder 1993).

Results from each of the five components are presented in Holder and colleagues (in press).

Three program elements in the northern California community were designed to increase public perceptions of increased DUI enforcement: (1) providing special BAC detection equipment to the local police as well as training in its use, (2) conducting DUI police enforcement checkpoints on a regular basis, and (3) increasing newspaper, television, and radio news coverage of local DUI enforcement. (See Voas [in press] for a detailed description of this component.) To increase news coverage of DUI enforcement and related policy actions, the Community Trials Project staff and volunteers in each of the experimental communities were intensively trained in media advocacy techniques. (See Treno and colleagues [1996] and Holder and Treno [in press] for a description and evaluation of media advocacy.)

### Testing the Intervention With SimCom

**Figure 5:**
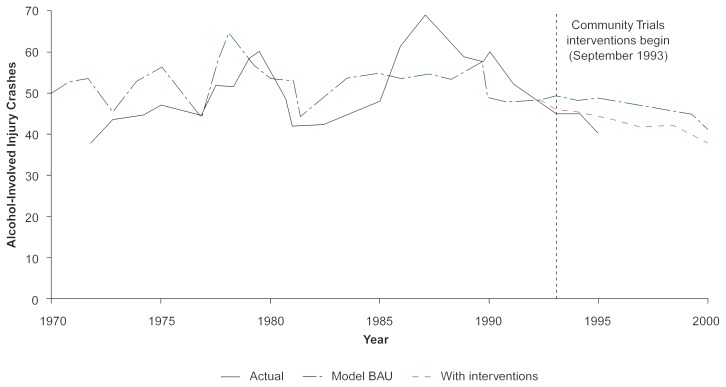
SimCom projections for alcohol-involved traffic crashes under two conditions: (a) business as usual (BAU), with no prevention interventions, and (b) with Community Trials drinking and driving policy interventions and a new California law concerning youth alcohol possession. Under the BAU projection, SimCom produced a continuation of the number of alcohol-involved crashes after 1993 which is higher than the actual, suggesting possible policy impacts. Entering the State and Community Trials interventions into SimCom produced results for 1993 to 2000 that extend the downward trend of the actual.

To validate the model against historical data, researchers first programmed SimCom with 1970 data from the northern California community. As part of the validation procedure, historical data after 1970 were not loaded into the model. Based on the 1970 figures, the SimCom model calculated the data values for the years 1971 to 1995. SimCom estimated per capita absolute alcohol consumption from 1970 through 1992 and the number of alcohol-involved injury crashes for the years 1972 to 1995. No model can recreate history perfectly, but the SimCom estimates demonstrated a good match with the actual data values for the two time periods (figures 3 and 5). Researchers therefore concluded that the model was valid.

SimCom then was loaded with data parameters that reflected new State laws governing alcohol consumption, along with parameters representing the news-coverage and law-enforcement components of the Community Trials intervention. The model projected alcohol-involved injury crashes under two conditions: (1) business as usual (BAU), with no prevention interventions, and (2) with Community Trials intervention (i.e., 1993 to 1995) complimented by State legislation. State legislation was implemented in January 1994 and specified a loss of driver’s license for any person under 21 years old who was caught drinking or in possession of alcohol. However, only 3 years of actual data from the Community Trials intervention (i.e., 1993 to 1995) were available for comparison with the model’s projections (see figure 5).

### Analysis of Results

Under the BAU conditions (i.e., the first condition), Sim-Com estimated a greater number of injury crashes than what actually occurred in the northern California site for the years 1993 to 1995 based on the situations that existed before 1993.

Under the Community Trials interventions (i.e., the second condition), SimCom produced forecasts for 1993 to 1995 that later closely matched actual data over the same period. These results demonstrate that SimCom was able to forecast accurately the local effect of community alcohol policy complimented by State policy directed at youthful drinkers. Statistical analyses of the northern California site, compared with its matched comparison site (which would have been influenced by State legislation but not the Community Trials intervention), showed significantly fewer crashes in the Trials community (see Voas et al. in press).

### Summary and Conclusions

Research rarely is packaged in formats that policymakers can use. Too frequently, research results of value to local decisionmakers do not reach them or, worse, are not understood and incorporated into policy decisions (see discussions by Langendorf 1985 and Edwards et al. 1994). The Community Trials test of SimCom illustrates how a computer model can be used to assist local planning. SimCom and similar computer models are easier than many other research techniques for nonresearchers to understand, because the model can be explained with simple flow charts rather than with complicated statistics. The mathematics driving the model is hidden beneath the surface logic, leaving the “bottom line” readily accessible.

Applying any computer model to a community context raises some important issues. The results from computer simulation always reflect the limits of our understanding about the system under study (i.e., it is not perfect). Computer model output should be used to assist policy formulation, not to make final decisions. In addition, a computer model may prove to be more complex and present more data collection requirements than can actually be accomplished in current prevention practice. The complexity of a sophisticated, computer-based model requires that model designers and community participants communicate well with each other to design the model and interpret the results in a useful manner. Furthermore, the information gleaned from computer models must be helpful to those who need it most: community prevention planners. Fortunately, to understand computer projections, prevention professionals and community planners need not evaluate each assumption from research and databases outside their community that go into developing the model.

The future is always uncertain, and computer models cannot account for unexpected influences. For example, any model’s predictions about alcohol-involved traffic crashes would not be able to account for a sudden gasoline price increase that would reduce all driving, including drinking and driving. In practice, the use of computer models remains on the cutting edge of prevention policy development. Current State and local planning of prevention programs typically requires a demonstration of potential benefits. Although local Community Trials project staff used the SimCom model of the northern California Community Trials project to give local policymakers an idea of the potential effectiveness of the prevention strategies, the model leaves room for improvement in estimating policy outcomes. The challenge for alcohol researchers is to provide alcohol prevention planners with accurate, reliable tools, such as computer simulation. With further refinement, computer models will no doubt become key components of community efforts to prevent alcohol-related problems.

## Footnotes

1 The rationale for this model is described by Holder (in press *a*,*b*).

2 Multivariate statistical techniques are those that focus on sets of outcome variables rather than on a single outcome variable.

3 The seven age groups are as follows: 13–17, 18–20, 21–24, 25–34, 35–49, 50–64, and 65 and older.

4 Alcohol-involved injury crashes are traffic crashes involving a driver or pedestrian under the influence of alcohol that result in injuries.
